# Diagnosis and treatment recommendations for glucose transporter 1 deficiency syndrome

**DOI:** 10.1007/s12519-024-00864-5

**Published:** 2025-01-02

**Authors:** Mei-Jiao Zhang, De Wu, Li-Fei Yu, Hua Li, Dan Sun, Jian-Min Liang, Xiao-Peng Lu, Rong Luo, Qing-Hui Guo, Rui-Feng Jin, Hong-Wei Zhang, Ge-Fei Lei, Ruo-Peng Sun, Man Wang, You-Feng Zhou, Ying-Yan Wang, Ji-Hong Tang, Ying Hua, Xu-Lai Shi, Xiao-Ming Liu, Xiu-Yu Shi, Guang Yang, Hua Wang, Feng Gao, Tian-Ming Jia, Ji-Wen Wang, Jian-Xiang Liao, Xin-Hua Bao

**Affiliations:** 1 The First Hospital of Peking University, Beijing, China; 2 The First Affiliated Hospital of Anhui Medical University, Anhui, China; 3 Children’s Hospital of Fudan University, Shanghai, China; 4 Guangdong Sanjiu Brain Hospital, Guangdong, China; 5 Huazhong University of Science and Technology Tongji Medical College Affiliated Wuhan Children’s Hospital, Wuhan, China; 6 Department of Pediatric Neurology, Children’s Medical Center, The First Hospital of Jilin University, Changchun, 130021 Jilin China; 7 Children’s Hospital of Nanjing Medical University, Nanjing, China; 8 Second Hospital of West China of Sichuan University, Sichuan, China; 9 The Second Hospital, Cheeloo College of Medicine, Shandong University, Shandong, China; 10 Children’s Hospital Affiliated to Shandong University, Shandong, China; 11 Qilu Hospital of Shandong University, Shandong, China; 12 Shanghai Deji Hospital, Shanghai, China; 13 Fujian Children’s Hospital (Fujian Branch of Shanghai Children’s Medical Center), College of Clinical Medicine for Obstetrics & Gynecology and Pediatrics, Fujian Medical University, Fujian, China; 14 Shanghai Children’s Medical Center, Shanghai Jiao Tong University School of Medicine, Shanghai, China; 15 Children’s Hospital of Soochow University, Jiangsu, China; 16 Wuxi Children’s Hospital, Jiangsu, China; 17 The Second Affiliated Hospital and Yuying Children’s Hospital of Wenzhou Medical University, Zhejiang, China; 18 Xuzhou Children’s Hospital, Jiangsu, China; 19 General Hospital of the Chinese People’s Liberation Army, Beijing, China; 20 Shengjing Hospital of China Medical University, Liaoning, China; 21 Children’s Hospital affiliated to Zhejiang University School of Medicine, Hangzhou, China; 22 The Third Affiliated Hospital of Zhengzhou University, Henan, China; 23 Shenzhen Children’s Hospital, Shenzhen, China

**Keywords:** Consensus, Diagnosis, Glut1DS, Treatment

## Abstract

**Background:**

Glucose transporter 1 deficiency syndrome (Glut1DS) was initially reported by De Vivo and colleagues in 1991. This disease arises from mutations in the *SLC2A1* and presents with a broad clinical spectrum. It is a treatable neuro-metabolic condition, where prompt diagnosis and initiation of ketogenic dietary therapy can markedly enhance the prognosis. However, due to its rarity, Glut1DS is susceptible to misdiagnosis or missed diagnosis, which can lead to delayed treatment and irreversible dysfunction of the central nervous system. To promote diagnostic awareness and effective treatments, the recommendations for diagnosis and treatment have been developed.

**Methods:**

The panel on Glut1DS included 28 participants from the members of the Ketogenic Diet Professional Committee of the Chinese Epilepsy Association and Chinese experts with extensive experience in managing Glut1DS. All authors extensively reviewed the literature, and the survey results were discussed in detail over several online meetings. Following multiple deliberative sessions, all participants approved the final manuscript for submission.

**Results:**

Early diagnosis and timely treatment of Glut1DS are crucial for improving prognosis. Physicians should be alert to suspiction of this disease if the following clinical manifestations appear: seizures, episodic or persistent movement disorders (often triggered by fasting, fatigue, or exercise), delayed motor and cognitive development. Characteristic clinical presentations may include seizures combined with movement disorders, episodic eye-head movements, and paroxysmal exercise-induced dyskinesia (PED). In these cases, genetic testing should be promptly completed, and a lumbar puncture should be performed if necessary. The ketogenic diet is internationally recognized as the first-line treatment; the earlier it is started, the better the prognosis. It can effectively control seizures and improve motor disorders. Antiepileptic drug treatment is generally ineffective or provides limited symptom improvement before starting the ketogenic diet.

**Conclusion:**

The recommendations provide clinicians with a relatively systematic guide for the rapid identification, diagnosis, and timely treatment of Glut1DS.

**Supplementary Information:**

The online version contains supplementary material available at 10.1007/s12519-024-00864-5.

## Introduction

Proper development and function of the human brain critically depend on a steady supply of glucose. Glucose transporter type 1 deficiency syndrome (Glut1DS), also known as De Vivo syndrome, is caused by a heterozygous pathogenic variant in the *SLC2A1*, which encodes the protein glucose transporter type 1 (Glut1). A deficiency in Glut1 impairs glucose transport into the brain across the blood–brain barrier, resulting in a series of symptoms related to cerebral energy deprivation [1]. Most Glut1DS cases are sporadic, with about 90% resulting from de novo heterozygous pathogenic variants in *SLC2A1*, following an autosomal dominant inheritance pattern. A smaller number of cases follow an autosomal recessive inheritance pattern. The clinical manifestations of Glut1DS vary widely in severity and often evolve with age. Early application of ketogenic diet therapy (KDT) can significantly enhance the prognosis for individuals with Glut1DS. Since its first description by De Vivo et al. in 1991 [1], a gradual increase in the number of reported cases of Glut1DS were found worldwide [2–6]. In 2020, Klepper et al. [7] published updated guidelines and recommendations by the Glut1DS International Research Group. Alongside many recent international researches [8–12], a Chinese multicenter clinical study was conducted [13]. In this cohort of 19 patients, the most common initial symptom was epileptic seizures (68%). Cerebrospinal fluid (CSF) glucose levels were below 2.2 mmol/L in 94.1% of cases, and the CSF glucose/blood glucose ratio was less than 0.45. After treatment with a ketogenic diet, 94.7% of epileptic seizures were effectively controlled, and 57.9% of motor disorders were effectively improved.

As a rare neurological disease, Glut1DS is susceptible to misdiagnosis or underdiagnosis, potentially leading to delayed intervention and irreversible harm to patients. Consequently, the Ketogenic Diet Professional Committee of the Chinese Epilepsy Association developed this consensus based on an extensive literature review and an experts' survey on Glut1DS diagnosis and treatment.

### Consensus recommendations

#### Epidemiology and pathogenesis

Larsen et al. reported that the frequency of *SLC2A1* mutations in epilepsy is about 1 in 80,000 [14]. Epidemiological investigations into neurological developmental disorders arising from de novo single-gene mutations in European and American countries estimate the incidence of Glut1DS to be 1.65–4.13 per 100,000 live births [15, 16]. Retrospective analyses in Denmark and Australia estimate the prevalence of Glut1DS to be 1 in 83,000 and 1 in 90,000, respectively. However, these figures are likely underestimates due to the potential for underdiagnosis [7].

Glucose is an essential metabolic fuel for the brain. Glut1, a key transmembrane protein, is predominantly expressed in endothelial cells and astrocytes, which constitute the blood–brain barrier in the central nervous system. It facilitates the transport of glucose from the bloodstream into the brain. Mutations in the *SLC2A1* can lead to decreased expression or dysfunction of Glut1, impairing glucose delivery to the brain for energy supply, leading to a series of neurological symptoms. In *SLC2A1* mutant mouse models [17], Glut1 deficiency due to *SLC2A1* haploinsufficiency can impede cerebral angiogenesis, significantly reducing the brain’s microvascular system without compromising the integrity of the blood–brain barrier. In addition to energy failure and angiogenesis abnormalities, cerebral glucose deficiency may compromise the pentose phosphate pathway and disrupt protein glycosylation, further contributing to the signs and symptoms of Glut1DS [18].

#### Clinical characteristics

The onset of Glut1DS can span from infancy to adulthood, with the neonatal period typically being asymptomatic. About 90% of cases are sporadic, with a minority having a family history. Symptoms tend to be mild or absent in parents who have heterozygous mutations [19, 20].

The clinical manifestations of Glut1DS are diverse and multifaceted, ranging from mild to severe. Symptoms usually present in infancy, including epileptic seizures, complex movement disorders, and delayed psychomotor development. The clinical features of Glut1DS are age-specific, with episodic eye-head movements and seizures often presenting in infancy, followed by developmental delays, movement disorders, and ataxia emerging later. In adolescent and adult patients, movement disorders are the most frequently reported symptoms [7, 20].

#### Epilepsy

Seizures are often the inaugural symptom of Glut1DS, affecting about 90% of patients and typically emerging before the age of 2 years, most commonly between 1 and 6 months [5]. Seizure types can vary with generalized seizures more prevalent than focal seizures. Generalized tonic–clonic seizures and absence seizures are the most common seizure types. Over two-thirds of patients experience two or more seizure types. Early-onset absence seizures and myoclonic-atonic seizures occurring before the age of 4 years may be linked to *SLC2A1* mutations. Epileptic spasms are also observed, though Lennox–Gastaut syndrome remains unreported [5, 7, 13, 19, 21]. Seizure frequency, severity, and types differ among individuals. Glut1DS should be suspected in any patients presenting with both epilepsy and movement disorders. Epilepsy is often the main clinical concern in infants and young children with Glut1DS, but tends to diminish or disappear in later childhood, adolescence, and adulthood. Although the most patients with Glut1DS experience drug-resistant epilepsy, KDT has shown significant efficacy.

#### Movement disorder

In early infancy, characteristic episodic eye-head movements are the second most common initial symptom. These episodes, also known as aberrant gaze saccades, are involuntary and brief multidirectional eye movements, usually conjugate, with concurrent head movements in the same direction. A study shows that aberrant gaze saccades present as an initial symptom in over one-third of patients with Glut1DS, highlighting their potential as an early diagnostic indicator; however, they are frequently overlooked by parents [4]. Occasionally, these eye-head movements are accompanied by cyanosis or apnea, leading to misdiagnosis as focal seizures. These symptoms are generally relieved by the age of 6 years. In later childhood, other episodic events with varying severity may occur, such as involuntary movements, ataxia, weakness, or paralysis, typically without consciousness impairment. In adults, symptoms tend to improve, exhibited by decreased frequency and severity. Additionally, some patients may experience paroxysmal non-motor events including migraine, behavioral disturbances, periodic vomiting, and somnolence [22–27].

Symptoms of movement disorders in Glut1DS range from mild to severe and may be constant, episodic, or fluctuating. These symptoms are often exacerbated by fasting and are alleviated postprandially. Persistent movement disorders encompass spasticity, ataxia, and dystonia, followed by chorea and tremors. A study [23] found that about 89% of patients exhibit various gait abnormalities, with spastic-atactic gait and ataxia being the most common. Ataxia typically becomes more pronounced during late infancy as children begin to stand and walk, primarily affecting the trunk. Choreoathetosis is typically mild, affecting the face and distal upper limbs, while terminal intention tremors are common and often concurrent with other signs of cerebellar dysfunction.

Myoclonus in Glut1DS can manifest as both epileptic and non-epileptic forms, with non-epileptic types being less common, such as startle-induced, action-induced, and positional myoclonus. About 75% of patients experience paroxysmal movement disorders, including episodic eye-head movements, paroxysmal exercise-induced dyskinesia (PED), paroxysmal kinesigenic dyskinesia, paroxysmal non-kinesigenic dyskinesia, episodic ataxia, and other paroxysmal events primarily manifesting as movement abnormalities or complex neurological symptoms [7, 13, 19, 21–27]. These movement disorders occur frequently, often provoked by fasting, exercise, and other triggers like emotional stress, fever, fatigue, sleep deprivation, temperature fluctuations, and certain medications.

#### Developmental and cognitive function

Most patients with Glut1DS have mild to severe intellectual disabilities, often correlating with the overall severity of the disease. Those with milder forms may possess normal cognitive abilities. Although patients can exhibit varying degrees of speech impairments, they typically adapt well in social and school environments, engaging positively with peers and displaying negligible signs of autism spectrum disorders. Notably, behavioral skills are more frequently impacted than speech, with pronounced defects in visual–spatial cognition and visual–motor abilities [19, 28].

#### Craniofacial features

Generally, the head circumference of patients with Glut1DS is smaller than that of healthy individuals. Microcephaly may present in infancy to varying degrees, often reflecting the clinical severity of the disorder, although it is not a consistent feature of Glut1DS [29]. A 3D facial analysis study of 11 Italian females aged 3–32 years with Glut1DS by Pucciarelli et al. [30] identified distinct craniofacial features in patients, such as a more anterior chin, an elongated mandibular body coupled with shorter rami, a reduced gonial angle, and smaller, downward-slanted eyes with a reduced intercanthal distance.

#### Atypical manifestations

Uncommon symptoms of Glut1DS include writing spasms, periodic generalized paralysis, parkinsonian symptoms, and nocturnal painful leg cramps. Some patients may experience alternating hemiplegia of childhood, hemiplegic migraine, periodic vomiting, stroke-like episodes with transient mild hemiparesis [31], dysarthria or aphasia, periodic weakness or fatigue, sleep disturbances, and somnolence. Other rare symptoms include hemolytic anemia associated with PED, hepatosplenomegaly, periventricular calcifications, cerebral atrophy, pseudo-hyperkalemia, cataracts, and retinal dysfunction [7, 19]. The mechanisms causing hemolytic anemia in this context are complex. Functional studies in Xenopus oocytes and human erythrocytes revealed that this mutation decreases glucose transport and causes a cation leak that alters intracellular concentrations of sodium, potassium, and calcium. Erythrocytes with an increased cation leak tend to swell and eventually undergo hemolysis [32].

#### Adult Glut1DS

Data on Glut1DS in adults is scarce. As patients age increase, the symptomatology often transitions from seizures typical in infancy or childhood to movement disorders, such as PED, which tend to emerge during adolescence or adulthood [7, 19].

In summary, the clinical manifestations of Glut1DS evolve with age. Episodic eye-head movements in infancy are highly indicative of Glut1DS. Other suggestive clinical features include episodic events of unknown cause at any age, absence seizure presenting before the age of 4 years, myoclonic-atonic epilepsy, drug-resistant epilepsy in children effectively controlled by KDT, and any unexplained movement disorders such as spasms, dystonia, and ataxia. Isolated clinical presentations, such as unexplained developmental delay, alternating hemiparesis, nonspecific episodic events, and stroke-like episodes [31], are rare in Glut1DS.

## Auxiliary examinations

### Electroenceph

Interictal electroencephalograms (EEGs) in Glut1DS patients of all age could be normal. However, different EEG abnormalities may emerge in certain age groups. Focal slow wave and epileptiform discharges are often seen in infants, while diffuse spike or sharp waves of 2.5–4.0 Hz may be observed in children over 2 years. A notable EEG feature of Glut1DS is the postprandial amelioration of the fasting-state EEG abnormalities [7, 19, 33].

### Cerebrospinal fluid

The hallmarks of Glut1DS are reduced cerebrospinal fluid (CSF) glucose levels (hypoglycorrhachia) alongside normal blood glucose levels. A lumbar puncture should be performed after 4–6 hours of fasting, with concurrent blood glucose measurements conducted preceding the procedure. The reference range of CSF glucose is age-dependent. CSF glucose levels in Glut1DS patients typically range from 0.9 to 2.9 mmol/L (16.2–52.0 mg/dL), consistently below the threshold of 3.3 mmol/L (60 mg/dL). The CSF glucose/blood glucose ratio ranges from 0.19 to 0.59 [5]. Most patients exhibit CSF glucose levels below 2.2 mmol/L (40 mg/dL) and a CSF glucose/blood glucose ratio below 0.4. In terms of diagnostic value, a reduction in absolute CSF glucose levels is a more reliable indicator than the CSF glucose/blood glucose ratio and correlates with symptom severity [7, 13, 19, 34]. Additionally, a normal or low CSF lactate supports the clinical diagnosis, while a low CSF lactate can rule out other diagnoses that might cause a low CSF glucose, such as mitochondrial disease and hexokinase 1 deficiency [35], where lactate levels are typically elevated. Recent studies have identified novel CSF markers, namely gluconic + galactonic acid, xylose-α1–3-glucose, and xylose-α1–3-xylose-α1–3-glucose, that are significantly decreased in patients with Glut1DS [18].

### Neuroimaging

While most Glut1DS patients have normal cranial magnetic resonance imaging (MRI) results, about one-quarter exhibit nonspecific abnormalities, such as high intensity of subcortical U-fibers on fluid-attenuated inversion recovery (FLAIR) sequences, expanded perivascular spaces, and delayed myelination [7, 11, 19]. A recent study involving three adults with late-onset Glut1DS revealed markedly reduced intensity in the bilateral caudate and lentiform nuclei on T1-weighted, T2-weighted, and T2 diffusion-weighted MRI, suggesting possible iron deposition [36]. Additionally, ^18^F-fluorodeoxyglucose positron emission tomography (^18^F-FDG-PET) typically demonstrates diminished thalamic metabolism and heightened basal ganglia metabolic activity in Glut1DS patients. Another recent study [37] found that the age-adjusted putamen/thalamus radioactivity ratio on PET can differentiate Glut1DS from epilepsy of unknown cause, with high sensitivity and specificity, which is crucial for accurate diagnosis. Magnetic resonance spectroscopy imaging has also been used to evaluate brain energy metabolism in individuals with Glut1DS, though further research is necessary to determine its diagnostic sensitivity and specificity.

### Genetic testing

Glut1DS predominantly follows an autosomal dominant inheritance pattern, with infrequent autosomal recessive cases. Among cases with positive genetic testing, 81%–89% of patients harbor de novo heterozygous mutations or rare homozygous mutations in the *SLC2A1* gene. About 11%–14% of patients are diagnosed through gene deletion/duplication analysis. Notably, *SLC2A1*-negative Glut1DS is reported in 5% to 15% of cases with a typical Glut1DS phenotype and low CSF glucose values worldwide. Therefore, the absence of an *SLC2A1* mutation does not exclude a Glut1DS diagnosis, even if other genetic defects have not been identified, as mutations could affect non-coding regions or downstream processes, such as Glut1 translation, transcription, activation, and transport. In general, there is a correlation between genotype and phenotype, with missense mutations often resulting in mild to moderate symptoms, while splice and nonsense mutations, as well as insertions, deletions, and exon deletions leading to moderate to severe symptoms. Complete gene deletions are associated with the most severe phenotypes [6, 7, 19, 38]. However, clinical heterogeneity exists within *SLC2A1* mutations, and the genotype–phenotype relationship is not entirely clear.

### Other laboratory studies

Beyond the central nervous system (CNS), Glut1 is also highly expressed on the membrane of erythrocytes. Consequently, the uptake of 3-O-methyl-D-glucose (3-OMG) by erythrocytes can indirectly reflect the function of Glut1 transporting glucose to the CNS. About 98.6% of Glut1DS patients diagnosed through genetic testing have reduced 3-OMG uptake, while around 1.4% of patients with missense mutations have normal uptake ability [39]. The uptake in Glut1DS patients ranges from 35 to 74% (with an average of 50%) compared to healthy individuals, offering diagnostic value [19, 39, 40]. However, this assay requires prompt completion after blood collection and specialized laboratory facilities equipped for radiotracer experiments, limiting its clinical application. An alternative approach is to express the patient's defective Glut1 in *Xenopus laevis* oocytes to evaluate its glucose uptake capacity, thus determining Glut1 functionality and disease severity. Quantitative assessment of Glut1 expression can be performed through techniques such as Western blotting [41], flow cytometry (METAglut1) [42, 43] and confocal immunofluorescence microscopy [21]. A prospective multicenter study showed that METAglut1 was 80% sensitive and > 99% specific for the diagnosis of Glut1DS [42]. Confocal immunofluorescence microscopy can help determine the localization of Glut1 and assess the pathogenicity of gene variants [21]. Gras et al. [44] reported that about 78% of Glut1DS patients exhibit at least a 20% (ranging from 23 to 59%) reduction in Glut1 expression on the surface of circulating erythrocytes, which may hold diagnostic relevance for Glut1DS. In addition, Cappuccio et al. indicated that plasma and urine metabolomics can reveal alterations in lipid and amino acid profiles in Glut1DS patients, suggesting potential impacts on mitochondrial physiology [45].

### Diagnosis and differential diagnosis

**Fig. 1:**
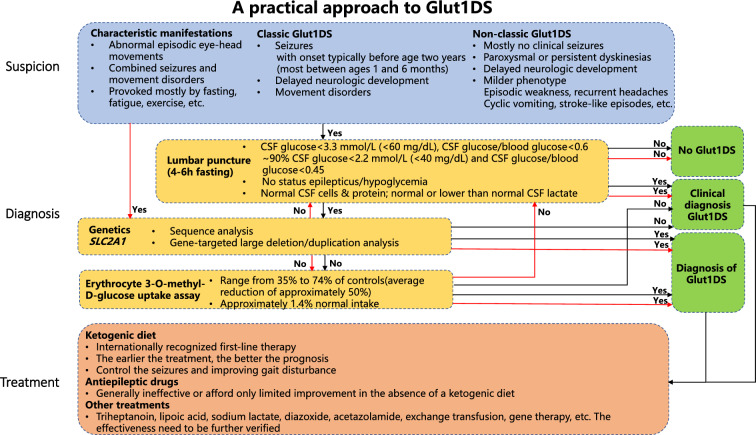
Flow chart of diagnosis and treatment of Glut1DS. *Glut1DS* glucose transporter 1 deficiency syndrome, *CSF* cerebrospinal fluid

Typically, Glut1DS can be diagnosed in patients presenting with characteristic clinical symptoms, low CSF glucose levels, and pathogenic *SLC2A1* variants (Fig. 1). In cases where patients display classic symptoms and low CSF glucose but lack an *SLC2A1* mutation, or have low CSF glucose and an *SLC2A1* mutation without classic symptoms, Glut1DS remains a highly probable diagnosis. Such scenarios necessitate the commencement of KDT. If KDT proves effective or there is a family history of Glut1DS, the diagnosis is further supported. If the patient has the characteristic clinical manifestations of Glut1DS and a pathogenic *SLC2A1* variant but has normal CSF glucose levels, or lacks typical symptoms but has either low CSF glucose or an *SLC2A1* mutation, a possible Glut1DS diagnosis is considered, and KDT should be tried [7].

The differential diagnosis of Glut1DS includes diseases causing low CSF glucose, epilepsy, movement disorders, and paroxysmal neurological symptoms [19, 46, 47]. Diseases that cause low CSF glucose, such as hypoglycemia, central nervous system infections, inherited metabolic disorders, subarachnoid hemorrhage, and status epilepticus, can often be differentiated based on their clinical manifestations. Additionally, two chronic neurodevelopmental disorders, *HK1* Variants and PURA (also referred to as Pur-alpha) syndrome, have been reported to present with low CSF glucose in some patients (HK1 variants) [35] or potentially in most patients (PURA syndrome) [48]. CSF lactate levels are found to be significantly higher than age-specific references values in individuals with HK1 variants, whereas patients with Glut1DS have low to normal CSF lactate levels. Genetic testing is essential for the differentiation of these diseases. Other diseases that need to be considered in the differential diagnosis of Glut1DS include opsoclonus-myoclonus syndrome, epileptic encephalopathy with developmental delay, familial epilepsy with autosomal dominant inheritance, and carbohydrate-responsive or preventable paroxysmal disorders, particularly those presenting with alternating hemiparesis, ataxia, cognitive dysfunction, seizures, and movement disorders.

## Treatment

### Ketogenic diet therapy

The current standard treatment for Glut1DS is the KDT, a high-fat diet that raises levels of ketone bodies in the blood, making them available to the brain. KDT should be initiated promptly, as it is highly effective in seizure control, often leading to seizure freedom, EEG normalization, and withdrawal of previously used antiepileptic drugs in most patients. KDT also improves movement disorders, cognitive impairment, and language problems in Glut1DS patients. Seizure control typically occurs within days to weeks of starting KDT, with earlier initiation leading to more significant benefits. Improvements in movement disorders typically take several months to become evident. Patients with higher CSF glucose/blood glucose ratios exhibit more favorable therapeutic outcomes. Early KDT initiation correlates with better Glut1DS prognosis [7, 19, 47, 49, 50]. Nonetheless, some reports indicate KDT ineffectiveness in seizure control, particularly in patients with late-onset seizures, delayed diagnosis, and late KDT initiation [13, 51].

KDT initiation and supervision in children with Glut1DS should adhere to the latest International Ketogenic Diet Study Group recommendations and domestic epilepsy treatment guidelines [52, 53]. KDT efficacy and adverse reactions warrant careful evaluation. The classic ketogenic diet, typically inducing higher ketosis levels, is recommended for younger children, especially infants and toddlers. The Modified Atkins Diet (MAD) may suit non-compliant children, adolescents, and adults. Low glycemic index treatment (LGIT) is not recommended due to its minimal ketone production. If tolerated, KDT should be maintained long-term, usually into adolescence or adulthood, although lifelong necessity remains uncertain. The international recommendations for the management of ketogenic diet therapy in adults with Glut1DS suggest that KDT may be both effective and safe, but further evidence-based research [54] is required. Monitoring adverse reactions, particularly carnitine deficiency, is crucial, and regular assessment of carnitine levels is recommended [7, 19, 47, 49].

### Anti-seizure medications

Data on the use of anti-seizure medications (ASMs) in Glut1DS is limited and contentious. A minority of patients with generalized epilepsy respond to conventional ASMs, and late-onset generalized tonic–clonic seizures may predict responsiveness to these medications. However, ASMs do not address the inherent metabolic defects in Glut1DS and could exacerbate potential adverse reactions associated with KDT. Thus, they are generally not recommended [7, 13, 19]. Medications such as ethosuximide, carbamazepine, oxcarbazepine, and zonisamide have shown ineffectiveness in this context. Carbonic anhydrase inhibitors may increase the risk of acidosis and urolithiasis, especially when combined with KDT. In vitro studies suggest that phenobarbital, diazepam, sodium valproate, hydrate chloral, methylxanthine derivatives, and ethanol may inhibit Glut1 function, while carbamazepine and phenytoin have no inhibitory effects. Phenytoin and its metabolic derivative, 5-(4-hydroxyphenyl)−5-phenylhydantoin, may enhance glucose transport by 30% to 60%, although the in vivo effects of this are unclear.

### Other treatments

1. Triheptanoin: Recent studies [55, 56] indicate that triheptanoin may reduce absence seizures in some Glut1DS patients; however, it does not significantly reduce overall seizure frequency and paroxysmal movement disorders in individuals who are not on a ketogenic diet;
2. Lipoic acid: Preliminary in vitro studies show that lipoic acid can enhance Glut1-mediated glucose transport. However, there is currently no clinical evidence to support this finding, and the oral dosage in patients may not reach the experimental levels needed for efficacy;
3. Sodium lactate: In certain clinical situations, lactate can provide energy to the brain. The proposed cellular mechanism involved astrocytes converting glucose into lactate through aerobic glycolysis, which is then shuttled as an energy source for neurons. A preliminary study suggests that sodium lactate, acting as an alternative energy source, may have a potential therapeutic effect in individuals with Glut1DS [57];
4. Diazoxide: There is potential for using diazoxide to increase blood sugar levels as an alternative approach to enhance cerebral glucose when KDT is ineffective or cannot be implemented [58];
5. Acetazolamide: A concise study of acetazolamide in Glut1DS epilepsy found that it decreased seizures in 76% of the patients, with 58% of all patients studied experiencing seizure reductions by more than half [59];
6. Exchange transfusion: The red blood cells of Glut1DS patients often have low glucose levels. Replacing these with normal donor red blood cells via exchange transfusion could enhance glucose transport from erythrocytes to neural cells [41].

### Management and prospects

Ongoing follow-ups are vital for Glut1DS patients due to symptoms evolution from predominant seizures in infancy to movement disorders in later life, necessitating personalized care. Cognitive impairment may persist throughout life. As patients approach adulthood, devising a comprehensive medical care strategy, including dietary therapy and transition from pediatric to adult care, becomes imperative. Long-term side effects of KDT, such as growth delays, kidney stones, and cardiovascular risks, require regular monitoring. Observational studies on KDT in adult patients, including those of reproductive age, are needed [7, 19].

Some clinical aspects of Glut1DS remain contentious, including the diagnostic cut-off value for CSF glucose, the significance of ambiguous *SLC2A1* variants, the diagnostic approach for asymptomatic individuals harboring *SLC2A1* mutations, and the use of KDT during pregnancy. Treatment strategies for atypical Glut1DS, especially for asymptomatic or late-onset cases, are yet to be established [7, 19]. Moreover, the necessity of newborn screening for this treatable disease warrants further investigation.

While KDT has proven effective for Glut1DS, exploring additional therapeutic avenues remains essential. Unresolved treatment issues include the efficacy and potential adverse effects of ASMs or certain compounds (such as acetazolamide, cannabidiol, ketone salts, and esters) when used alongside KDT. Furthermore, the management of paroxysmal episodes, dystonia, and dysarthria, which significantly impair patients' quality of life, has yet to be optimized. The appropriate duration of KDT in adult patients and strategies for monitoring and addressing its long-term adverse effects are still under investigation [7, 19]. Future research must also address the systemic implications of Glut1 deficiency, particularly in organs with high Glut1 expression, such as the heart, muscles, placenta, and retina [60]. Recent findings of compromised brain vascularization in Glut1DS mice models underscore the critical need for early diagnosis and intervention to prevent irreversible brain dysfunction. Future therapeutic strategies include supplementation of brain metabolic fuels, exploring *SLC2A1* therapy, implementing protein replacement therapy, trying mesenchymal stem cell -derived exosomes (MSC-Exo) therapy and developing small molecules to augment Glut1 expression and function [7, 61, 62].

## Supplementary Information

Below is the link to the electronic supplementary material.

Supplementary file 1 (PDF 3968 KB)

## Data Availability

Data sharing not applicable to this article as no datasets were generated or analysed during the current study.
