# Supplementary material for: Diagnosis and treatment recommendations for glucose transporter 1 deficiency syndrome
Source: World J Pediatr. 2025 Jan 2;21(2):149–58. doi: 10.1007/s12519-024-00864-5 (PMC11885374; doi:10.1007/s12519-024-00864-5)
Supplement: Supplementary file 1 — (PDF 3968 KB) [file 12519_2024_864_MOESM1_ESM.pdf]

SPRINGER NATURE

Change of authorship request form - Journals (pre-acceptance)

Section 1: Please provide the current title of manuscript

Manuscript ID no.: WJOP-D-24-00570-R1

Title: Diagnosis and treatment recommendations for glucose transporter 1 deficiency syndrome

Section 2: Please provide the previous authorship, in the order shown on the manuscript before the changes were introduced. Please indicate the corresponding author by adding (CA) behind the name.

	First name(s)	Family name	ORCID or SCOPUS id, if available
1st author	Mei-Jiao	Zhang	
2nd author	De	Wu	
3rd author	Li-Fei	Yu	
4th author	Hua	Li	
5th author	Dan	Sun	
6th author	Jian-Min	Liang	
7th author	Xiao-Peng	Lu	
8th author	Rong	Luo	
9th author	Qing-Hui	Guo	
10th author	Rui-Feng	Jin	

Please use an additional sheet if there are more than 10 authors.

SPRINGER NATURE Springer Nature is one of the world's leading global research, educational and professional publishers, created in May 2015 through the combination of Nature Publishing Group, Palgrave Macmillan, Macmillan Education and Springer Science+Business Media.

CS 扫描全能王
3亿人都在用的扫描App

Section 2: Please provide the previous authorship, in the order shown on the manuscript before the changes were introduced. Please indicate the corresponding author by adding (CA) behind the name.

	First name(s)	Family name	ORCID or SCOPUS id, if available
11th author	Hong-Wei	Zhang	
12th author	Ge-Fei	Lei	
13th author	Ruo-Peng	Sun	
14th author	Man	Wang	
15th author	You-Feng	Zhou	
16th author	Ying-Yan	Wang	
17th author	Ji-Hong	Tang	
18th author	Ying	Hua	
19th author	Xu-Lai	Shi	
20th author	Xiao-Ming	Liu	
21th author	Xiu-Yu	Shi	
22th author	Guang	Yang	
23th author	Hua	Wang	
24th author	Feng	Gao	
25th author	Tian-Ming	Jia	
26th author	Ji-Wen(CA)	Wang	
27th author	Jian-Xiang(CA)	Liao	
28th author	Xin-Hua(CA)	Bao	

CS 扫描全能王
3亿人都在用的扫描App

SPRINGER NATURE

Change of authorship request form - Journals (pre-acceptance)

Section 3: Please provide a justification for change. Please use this section to explain your reasons for changing the authorship of your manuscript, e.g. what necessitated the change in authorship? Please refer to the (journal) policy pages for more information about authorship. Please explain why omitted authors were not originally included and/or why authors were removed on the submitted manuscript.

No changes

Section 4: Proposed new authorship. Please provide your new authorship list in the order you would like it to appear on the manuscript. Please indicate the corresponding author by adding (CA) behind the name. <u>If the Corresponding Author has changed, please indicate the reason under section 3</u>.

	First name(s)	Family name (this name will appear in full on the final publication and will be searchable in various abstract and indexing databases)	Affiliated institute	E-mail address
1st author	Mei-Jiao	Zhang	The First Hospital of Peking University, Beijing, China	17839948947@163.com
2nd author	De	Wu	The First Affiliated Hospital of Anhui Medical University, Anhui, China	derk_wu@163.com
3rd author	Li-Fei	Yu	Children's Hospital of Fudan University, Shanghai, China	yulifei7711@163.com
4th author	Hua	Li	Guangdong Sanjiu Brain Hospital, Guangdong, China	lihua1051@163.com
5th author	Dan	Sun	Huazhong University of Science and Technology Tongji Medical College Affiliated Wuhan Children's Hospital, Wuhan, China	bloveriver@163.com
6th author	Jian-Min	Liang	Department of Pediatric Neurology, Children's Medical Center, The First Hospital of Jilin University, Changchun 130021, Jilin, China	liangjm@jlu.edu.cn
7th author	Xiao-Peng	Lu	Children's Hospital of Nanjing Medical University, Nanjing, China	lxp20071113@sina.com
8th author	Rong	Luo	Second Hospital of West China of Sichuan University, Sichuan, China	1109329811@qq.com
9th author	Qing-Hui	Guo	The Second Hospital, Cheeloo College of Medicine, Shandong University, Shandong, China	doctorguo@126.com
10th author	Rui-Feng	Jin	Children's Hospital Affiliated to Shandong University, Shandong, China	13953174587@163.com

Please use an additional sheet if there are more than 10 authors.

SPRINGER NATURE Springer Nature is one of the world's leading global research, educational and professional publishers, created in May 2015 through the combination of Nature Publishing Group, Palgrave Macmillan, Macmillan Education and Springer Science+Business Media.

CS 扫描全能王
3亿人都在用的扫描App

Section 4: Proposed new authorship. Please provide your new authorship list in the order you would like it to appear on the manuscript. Please indicate the corresponding author by adding (CA) behind the name. If the Corresponding Author has changed, please indicate the reason under section 3.

	First name(s)	Family name (this name will appear in full on the final publication and will be searchable in various abstract and indexing databases)	Affiliated institute	E-mail address
11th author	Hong-Wei	Zhang	Children's Hospital Affiliated to Shandong University, Shandong, China	zhw850510@163.com
12th author	Ge-Fei	Lei	Qilu Hospital of Shandong University, Shandong, China	leigefei@sdu.edu.cm
13th author	Ruo-Peng	Sun	Qilu Hospital of Shandong University, Shandong, China	ruopeng4813@sina.com
14th author	Man	Wang	Shanghai Deji Hospital, Shanghai, China	wangman168@sina.cn
15th author	You-Feng	Zhou	Fujian Children's Hospital（Fujian Branch of Shanghai Children's Medical Center）,College of Clinical Medicine for Obstetrics & Gynecology and Pediatrics, Fujian Medical University, Fujian, China	zyfux@yeah.net
16th author	Ying-Yan	Wang	Shanghai Children's Medical Center, Shanghai Jiao Tong University School of Medicine, Shanghai, China	wangyingyan@scmc.com.cn
17th author	Ji-Hong	Tang	Children's Hospital of Soochow University, Jiangsu, China	tjhzsh@126.com
18th author	Ying	Hua	Wuxi Children's Hospital, Jiangsu, China	huayingwxey@163.com

CS 扫描全能王
3亿人都在用的扫描App

19th author	Xu-Lai	Shi	The Second Affiliated Hospital and Yuying Children's Hospital of Wenzhou Medical University, Zhejiang, China	sunnywz48@sohu.com
20th author	Xiao-Ming	Liu	Xuzhou Children's Hospital, Jiangsu, China	xzlrlxm@163.com
21th author	Xiu-Yu	Shi	General Hospital of the Chinese People's Liberation Army, Beijing, China	shixiuyu@sina.com
22th author	Guang	Yang	General Hospital of the Chinese People's Liberation Army, Beijing, China	yanggg301@sina.com
23th author	Hua	Wang	Shengjing Hospital of China Medical University, Liaoning, China	shengjingwangh@163.com
24th author	Feng	Gao	Children's Hospital affiliated to Zhejiang University School of Medicine, Zhejiang, China	epilepsy@zju.edu.cn
25th author	Tian-Ming	Jia	The Third Affiliated Hospital of Zhengzhou University, Henan, China	jtm226@sina.com
26th author	Ji-Wen(CA)	Wang	Shanghai Children's Medical Center, Shanghai Jiao Tong University School of Medicine, Shanghai, China	wangjiwen@scmc.com.cn
27th author	Jian-Xiang(CA)	Liao	Shenzhen Children's Hospital, Shenzhen, China	liaojianxiang@vip.sina.com
28th author	Xin-Hua(CA)	Bao	The First Hospital of Peking University, Beijing, China	zwhang@pku.edu.cn

CS 扫描全能王
3亿人都在用的扫描App

Change of authorship request form - Journals (pre-acceptance)

Section 5: Author contribution, Acknowledgement and Disclosures. Please use this section to provide a new disclosure statement and, if appropriate, acknowledge any contributors who have been removed as authors and ensure you state what contribution any new authors made (if applicable per the journal or book (series) policy). Please ensure these are updated in your manuscript - after approval of the change(s) - as our production department will not transfer the information in this form to your manuscript.

New acknowledgements:

No changes

New Disclosures (financial and non-financial interests, funding):

No changes

New Author Contributions statement (if applicable per the journal policy):

Not applicable

State 'Not applicable' if there are no new authors.

SPRINGER NATURE Springer Nature is one of the world's leading global research, educational and professional publishers, created in May 2015 through the combination of Nature Publishing Group, Palgrave Macmillan, Macmillan Education and Springer Science+Business Media.

CS 扫描全能王
3亿人都在用的扫描App

Change of authorship request form - Journals (pre-acceptance)

Section 6: Declaration of agreement. *All* authors, unchanged, new and removed *must* sign this declaration.
(NB: Please print the form, (docu)-sign and return/upload a scanned copy. Please note that signatures that have been inserted as an image file are acceptable as long as it is handwritten. Typed names in the signature box are unacceptable.) *** Please delete as appropriate. Delete all of the bold if you were on the original authorship list and are remaining as an author.**

	First name	Family name		Signature	Date
1st author	Mei-Jiao	Zhang	I agree to the proposed new authorship shown in section 4 /and the proposed change in corres ponding author	Xinhua Bao	2024.11.8
2nd author	De	Wu	I agree to the proposed new authorship shown in section 4 /and the proposed change in corres ponding author	Xinhua Bao	2024.11.8
3rd author	Li-Fei	Yu	I agree to the proposed new authorship shown in section 4 /and the proposed change in corres ponding author	Xinhua Bao	2024.11.8
4th authors	Hua	Li	I agree to the proposed new authorship shown in section 4 /and the proposed change in corres ponding author	Xinhua Bao	2024.11.8
5th author	Dan	Sun	I agree to the proposed new authorship shown in section 4 /and the proposed change in corres ponding author	Xinhua Bao	2024.11.8
6th author	Jian-Min	Liang	I agree to the proposed new authorship shown in section 4 /and the proposed change in corres ponding author	Xinhua Bao	2024.11.8
7th author	Xiao-Peng	Lu	I agree to the proposed new authorship shown in section 4 /and the proposed change in corres ponding author	Xinhua Bao	2024.11.8

CS 扫描全能王
3亿人都在用的扫描App

Change of authorship request form - Journals (pre-acceptance)

	First name	Family name		Signature	Date
8th author	Rong	Luo	I agree to the proposed new authorship shown in section 4 /and the proposed change in corres ponding author	Xinhua Bao	2024.11.8
9th author	Qing-Hui	Guo	I agree to the proposed new authorship shown in section 4 /and the proposed change in corres ponding author	Xinhua Bao	2024.11.8
10th author	Rui-Feng	Jin	I agree to the proposed new authorship shown in section 4 /and the proposed change in corres ponding author	Xinhua Bao	2024.11.8

Please use an additional sheet if there are more than 10 authors.

In case of author collaborations with formal agreement:

	Name of consortium/consortia	First name	Family name		Signature	Date
Representative/legal guarantor				I agree to the proposed new authorship shown in section 4 /and the proposed chang e in corresponding author		

Both added/removed authors should complete the information in the first table under Section 6.

---- End of form ----

SPRINGER NATURE Springer Nature is one of the world's leading global research, educational and professional publishers, created in May 2015 through the combination of Nature Publishing Group, Palgrave Macmillan, Macmillan Education and Springer Science+Business Media.

CS 扫描全能王
3亿人都在用的扫描App

Section 6: Declaration of agreement. All authors, unchanged, new and removed must sign this declaration.(NB: Please print the form, (docu)-sign and return/upload a scanned copy. Please note that signatures that have been inserted as an image file are acceptable as long as it is handwritten. Typed names in the signature box are unacceptable.) * Please delete as appropriate. Delete all of the bold if you were on the original authorship list and are remaining as an author.

	First name	Family name		Signature	Date
11th author	Hong-Wei	Zhang	I agree to the proposed new authorship shown in section 4 /and the proposed change in corresponding author	Xinhua Bao	2024.11.8
12th author	Ge-Fei	Lei	I agree to the proposed new authorship shown in section 4 /and the proposed change in corresponding author	Xinhua Bao	2024.11.8
13th author	Ruo-Peng	Sun	I agree to the proposed new authorship shown in section 4 /and the proposed change in corresponding author	Xinhua Bao	2024.11.8
14th author	Man	Wang	I agree to the proposed new authorship shown in section 4 /and the proposed change in corresponding author	Xinhua Bao	2024.11.8
15th author	You-Feng	Zhou	I agree to the proposed new authorship shown in section 4 /and the proposed change in corresponding author	Xinhua Bao	2024.11.8
16th author	Ying-Yan	Wang	I agree to the proposed new authorship shown in section 4 /and the proposed change in corresponding author	Xinhua Bao	2024.11.8
17th author	Ji-Hong	Tang	I agree to the proposed new authorship shown in section 4 /and the proposed change in corresponding author	Xinhua Bao	2024.11.8
18th author	Ying	Hua	I agree to the proposed new authorship shown in section 4 /and the proposed change in corresponding author	Xinhua Bao	2024.11.8

CS 扫描全能王
3亿人都在用的扫描App

19th author	Xu-Lai	Shi	I agree to the proposed new authorship shown in section 4 /and the proposed change in corresponding author	Xinhua Bao	2024.11.8
20th author	Xiao-Ming	Liu	I agree to the proposed new authorship shown in section 4 /and the proposed change in corresponding author	Xinhua Bao	2024.11.8
21th author	Xiu-Yu	Shi	I agree to the proposed new authorship shown in section 4 /and the proposed change in corresponding author	Xinhua Bao	2024.11.8
22th author	Guang	Yang	I agree to the proposed new authorship shown in section 4 /and the proposed change in corresponding author	Xinhua Bao	2024.11.8
23th author	Hua	Wang	I agree to the proposed new authorship shown in section 4 /and the proposed change in corresponding author	Xinhua Bao	2024.11.8
24th author	Feng	Gao	I agree to the proposed new authorship shown in section 4 /and the proposed change in corresponding author	Xinhua Bao	2024.11.8
25th author	Tian-Ming	Jia	I agree to the proposed new authorship shown in section 4 /and the proposed change in corresponding author	Xinhua Bao	2024.11.8
26th author	Ji-Wen(CA)	Wang	I agree to the proposed new authorship shown in section 4 /and the proposed change in corresponding author	Xinhua Bao	2024.11.8
27th author	Jian-Xiang(CA)	Liao	I agree to the proposed new authorship shown in section 4 /and the proposed change in corresponding author	Xinhua Bao	2024.11.8
28th author	Xin-Hua(CA)	Bao	I agree to the proposed new authorship shown in section 4 /and the proposed change in corresponding author	Xinhua Bao	2024.11.8

扫描全能王
3亿人都在用的扫描App

clarification

I certify that each author agrees with the current order of identity.

Xinhua Bao

2024. 11. 8

扫描全能王
3亿人都在用的扫描App
